# Bonded labour and donkey ownership in the brick kilns of India: A need for reform of policy and practice

**DOI:** 10.1017/awf.2023.1

**Published:** 2023-01-27

**Authors:** Laura M Kubasiewicz, Tamlin Watson, Caroline Nye, Natasha Chamberlain, Ramesh K Perumal, Ramesh Saroja, Stuart L Norris, Zoe Raw, Faith A Burden

**Affiliations:** 1 The Donkey Sanctuary, Sidmouth, Devon EX10 0NU, UK; 2 Centre for Rural Policy Research, University of Exeter, Exeter, UK; 3 Donkey Sanctuary Welfare Association, Ahmedabad, India

**Keywords:** animal welfare, bonded labour, *Equus asinus*, one welfare, sustainable development, working donkeys

## Abstract

Slavery, in the form of ‘debt-bondage’, is rife in Indian brick kilns, where the enforcement of labour laws is poor. Working equids support brick-kiln workers by transporting raw bricks into the kilns, but the situation of equids and their owners within the brick kilns is relatively unknown. We describe the welfare of donkeys (*Equus asinus*) owned under conditions of debt-bondage, examine the links between owner and donkey behaviour, and outline the living conditions of both donkeys and humans working in the brick kilns of Gujarat, India. We then explore the unique experience of debt-bondage by donkey owners, compare migration trends to those of non-donkey-owning workers and assess impacts on their children’s education. The physical and behavioural conditions of donkeys reflected that of their owners, creating negative feedback loops and potentially reducing productivity. All donkey owners experienced debt-bondage and were particularly vulnerable to unexpected financial loss. Donkey owners, unlike non-owners, migrated within their home state, enabling their children to attend school. Our work highlights the need for policy reform within the brick-kiln industry to acknowledge the pivotal role of working donkeys in supporting human livelihoods.

## Introduction

An estimated 40 million people globally are enslaved within systems of modern slavery (Anti-Slavery International 2020). Over half of these work within bonded labour, which occurs most frequently in South Asia. Bonded labour occurs when: “…a person is forced to use their physical labour to pay off a debt. They are forced into working for little or no pay, with no control over their debt and the value of their work invariably becomes greater than the original sum of money borrowed” (Anti-Slavery International 2020).

According to John (2018), a presumption exists that “caste-based occupations and bonded labour are natural ingredients to the Indian brick-kiln industry” (ibid; p 13) without whom the estimated output of 700–800 million bricks a year would be impossible. Despite implementation of the Bonded Labour System (Abolition) Act in 1976, debt-bondage is now considered endemic in the brick-kiln industry in India (Anti-Slavery International 2017).

The Indian brick-kiln industry is officially regulated by several laws, including the Minimum Wage Act (1948), which permits regional governments to set minimum wages for different skill levels, industries, and job roles within their constituency. Enforcement of these laws is, however, notoriously poor. This is particularly true in the unorganised sector, which consists of privately owned small businesses engaged in the sale or production of goods (Government of India 2008), as these businesses are widely distributed and difficult to monitor (Gupta 2003; PCLRA 2012). In practice, brick-kiln workers may earn less than the threshold for extreme poverty (Guérin *et al.* 2007; Anti-Slavery International 2017), defined by the World Bank as an individual earning less than $US1.90 per day (Ferreira *et al.* 2015).

Wages in the brick kilns are usually paid to one ‘officially’ employed male worker, whilst work is actually conducted by this worker and his spouse (known as ‘jodi labour’) or by the whole family unit (Guérin *et al.* 2007). This arrangement results in ‘invisible workers’ who are unregistered and ineligible for employee protection (Anti-Slavery International 2017), whilst having no freedom to seek paid work elsewhere (Mazumdar *et al.* 2015). In the same regard, whilst the latest estimate for the number of equids worldwide is 116 million (FAO 2019), this figure is likely to be a gross underestimate due to a lack of official registration or reporting of domestic animals (Norris *et al.* 2021), and the role of equids in supporting human livelihoods is poorly acknowledged in regional, national or global policy (Valette 2015).

Approximately 380,000 equids support the work of brick loaders in the Indian brick kilns, where they transport moulded bricks into the kilns (Mitra & Valette 2017). Equid ownership is highly ingrained in the culture of particular Scheduled Castes in India (Watson *et al.* 2020), and often provides the main income source and only route out of extreme poverty for these communities (Kandpal *et al.* 2014; Geiger *et al.* 2020). For equid owners, human and animal exploitation and suffering are often intertwined (DeMello 2012), as the “systems limiting human economic and social mobility… also work to oppress and restrict animals” (Watson *et al.* 2020). For equid owners, the pressure to earn often results in their equids being overworked (Zaman *et al.* 2014), creating and exacerbating physical issues and psychological problems such as depression and unresponsiveness (Burn *et al.* 2010a). Working mules in Egyptian and Nepalese brick kilns display poor behavioural welfare when handled aggressively (Ali *et al.* 2019; Norris *et al.* 2020), which may also be symptomatic of the socio-economic pressures experienced by the owner, although a direct causal link has not been identified.

The brick kilns in Gujarat operate seasonally for six to eight months, from approximately November, during the dry season (Watson *et al.* 2020). Brick-kiln workers tend to be migrants, travelling within and between states, and internationally (PCLRA 2012; Mitra & Valette 2017). The current policy framework within India mandates universal coverage for education and health. In practice, however, few mechanisms exist to direct these services towards seasonal migrants (Wolfston 2019) as neither the source nor destination state take responsibility for their provision (Smita 2008). Over half of the working equids in India are located in the northern states, particularly Uttar Pradesh (Singha *et al.* 2020), which acts as the main equid trading hub from which equids and their owners travel to brick kilns in more prosperous states such as Gujarat (Malik *et al.* 2012; Mitra & Valette 2017). In recent years, however, equid movement has been curtailed by the re-emergence of glanders, a highly infectious and often fatal zoonotic disease (Government of India 2016; Singha *et al.* 2020), which may have affected the seasonal migration of equid-owning kiln workers, although evidence for this change is currently lacking.

Literature on the economic situation of brick-kiln workers rarely references the role of equids. Although the presence of equids is briefly mentioned in relation to the actual (Ercelawn & Nauman 2004) or state minimum (John 2014) earnings of their owners, to our knowledge there are no assessments of whether state minimum wages are met for equid owners, nor details of the specific impacts of equid ownership on debt-bondage arrangements.

In this study, we describe the welfare of donkeys (*Equus asinus*) owned under conditions of debt-bondage in the brick kilns of Gujarat, India. Following the identification of ‘shared suffering’ between humans and donkeys in the current cohort (Watson *et al.* 2020), we then identify potential links between human and donkey behaviour, and review the living conditions of both humans and donkeys. In a separate analysis of the current dataset, we outlined the impact of donkey ownership on income poverty, providing a relative comparison of wealth between donkey-owning and non-owning kiln workers (Kubasiewicz *et al.* 2022). Here, we provide a follow-up piece, outlining the position of donkey owners in relation to minimum wage thresholds and their unique experiences of debt-bondage. Finally, we compare levels of migration between donkey owners and non-donkey-owning workers, and assess the impacts of the potentially higher intra-state migration of donkey owners on their children’s school attendance.

## Materials and methods

Fieldwork was conducted between 30th April and 14th May 2018 in 14 brick kilns situated in and around Ahmedabad, Gujarat state, India. Specific brick kilns were selected based on both accessibility and permission from the kiln owner, which was gained due to the existing presence of Donkey Sanctuary within the kilns. Donkey Sanctuary India (DSI; now named Donkey Sanctuary Welfare Association) provided logistical support and interpretation services throughout the study. DSI provided ongoing veterinary interventions to participants within the kilns accessed, and we acknowledge the potential influence of this on some responses during interviews. Gaining access to sites, however, would not have been possible without their assistance. As the study did not include an exploration of Non-Government Organisation (NGO) assistance, the potential for bias is not likely to have been substantial.

We classified participants as ‘donkey owners’ (brick transporters, with donkeys working as pack animals), ‘thekedars’ (contractors in a supervisory role), ‘non-owners’ (brick moulders, stackers and firemen) and kiln owners. Thekedars oversee a specific job role within the kilns; in this case the brick transporters. All thekedars also owned and worked with donkeys as brick transporters.

### Ethical considerations

The study was conducted in accordance with the Declaration of Helsinki and the protocol approved by the Ethics Committee of The Donkey Sanctuary, Project Number 2018-VOD-INDIA. Fieldwork was conducted between 0800 and 1300h after kiln work ended. Researchers ensured that people had the chance to rest, eat and recover and that donkeys had been fed and watered before conducting assessments and interviews. All participants provided recorded verbal consent, were given the right to withdraw within two weeks of data collection and were anonymised in accordance with data protection guidelines.

As principal researchers are from a different cultural background to participants, there was potential for interview questions and themes to differ from the participants’ perspectives, or for responses to be misinterpreted. To reduce this potential bias, interview topics and questions were developed in collaboration with colleagues at Donkey Sanctuary India and adapted throughout the study; responses were discussed after each interview to ensure a shared understanding. Participants were recruited based on their availability at the time of the visit, willingness to participate and their job role in order to obtain a stratified sample of job roles within the kiln environment. There is, therefore, the potential for bias and the sample may not fully represent the population as a whole, so generalisations based on the current findings should be made with caution.

### Data collection

Donkey welfare was assessed by trained welfare assessors using the EARS (Equid Assessment Research and Scoping) tool (Raw *et al.* 2020). The EARS tool allows bespoke protocols to be developed by selecting appropriate questions to suit a particular context. Question selection for the current research piece included both direct assessment of the equid and questions to ask the equid owner (see Table S1). All donkeys belonging to a specific owner were tethered in a line, so sampling was conducted by assessing each donkey from left to right in the line, regardless of the condition of the equid. Sample size was dictated by the time needed to complete a questionnaire and interview the owner.

A living conditions assessment was carried out at each kiln to collect data on the kiln environment and access to resources for both humans and donkeys (see Table S2). Data were collected through observation of the kiln by principal researchers or, where resources (such as water points) were not visible, participants were asked to describe their location.

Questionnaires gathered data on wages and production; bonded labour (advanced payment); and childrens’ education, where school-age children are classed as those between 6 and 14, who are eligible for free and compulsory education under the Right of Children to Free and Compulsory Education Act (RTE; Government of India 2021) (see Table S3). Participants were able to opt out of any questions they did not wish to answer therefore sample sizes may differ from the total cohort in some cases.

Semi-Structured Interviews (SSIs) explored the broad topics of bonded labour; savings and debt; migration; and children’s education (see Table S4). The semi-structured approach enabled new topics to arise during discussion. Interviews lasted from 10 to 50 min, averaging 35 min, were audio-recorded and the English interpretation was later transcribed *verbatim.*

Questionnaires and SSIs were carried out consecutively with each participant, except when a participant did not have time or did not wish to complete an interview. Both were conducted by principal researchers in English and interpreted to and from Hindi *in situ* by Indian colleagues from Donkey Sanctuary India. Hindi was selected as the most likely common language between interpreters and participants, as we expected a proportion of participants to originate from outside Gujarat. Whilst all participants were able to converse in Hindi, we acknowledge the potential for misinterpretation in cases where Hindi was not their first language.

Field notes were also collected throughout, where principal researchers recorded observations within the kilns that were relevant to the research topics or may have introduced bias (e.g., the presence of a superior during an interview), explanations of events by colleagues from DSI, or summaries of interview responses that were of particular interest or prompted a new research topic or theme.

Questionnaire, EARS and living conditions data were recorded digitally using Open Data Kit (ODK) collect (Hartung *et al.* 2010) and uploaded to a server once the device was connected to the internet.

### Data analysis

EARS assessment, living conditions assessment, and questionnaire results are presented as descriptive statistics unless otherwise state below. As participants were not required to answer a question if they did not wish to do so, sample sizes may differ per question.

Whilst some of the variables presented are not normally distributed, our sample is relatively small and we did not want to discount the importance of extreme values. As such, we present descriptive statistics as the mean and standard deviation (see Lydersen 2020).

Quantitative data were analysed in R (V 3.6.1; R Core Team 2019) using R.Studio (V 1.2.5001; Racine 2012).

#### Linking human and donkey behaviour

To explore potential links between human and donkey behaviour, responses to the EARS questions ‘Owner’s/user’s/handler’s interaction when holding the equid’ and ‘response to observer approach’ were classified as ‘positive/neutral’ or ‘negative’ (see Table S3). We then calculated whether more or fewer equids that experienced negative interactions with their owners displayed negative responses to approach by an EARS assessor than those that experienced positive interactions.

#### Analysis of wages and production

Participants were asked to report their family’s wages from brick-kiln work inclusive of money deducted, saved and spent. To allow for triangulation, we also calculated daily earnings based on piece rates, number of bricks moved per day (in total and per donkey) and number of donkeys worked per day, where data were available.

Mean daily wages per person were calculated by dividing family daily wages by the number of adults in the household at the kiln. The situation is, however, complex, as some adults who travel to the kilns with their family do not work and, therefore, do not contribute towards their family’s income, despite being reliant and counted within the adults in their household. To provide a dataset for wages per person with the highest level of certainty, we limited data to family units consisting of no more than two adults. Wages were then compared to the following established wage thresholds; the World Bank international poverty line of $US1.90 per day (Ferreira *et al.* 2015), following a conversion to Rs133 using the 2018 exchange rate of Rs70 per $US1; and the minimum wage for ‘bhartiwala’ (raw brick carrier) in Gujarat of Rs276 (basic rate) to Rs312 (inclusive of special allowance). No minimum wage is set for thekedars, specifically, therefore the rate for ‘accountant supervisors’ and ‘miscellaneous labour’ of 293 (basic rate) to 329 (inclusive of special allowance) was used. The special allowance refers to either an amount paid in addition to the basic salary to cover expenses, or any additional salary provided by the employer to meet the specific requirements of a role, as agreed with the employee (Government of India 1961).

Minimum wages for Gujarat are set for specific job roles within the kilns, per day. To avoid exploitation by employers, the government includes a stipulation that wages paid via piece rates (i.e., set payments per amount of work) must take productivity into account, so that employees can make at least the minimum daily wage for their job role (Government of India, Ministry of Labour 2020). For brick loaders, this ‘production norm’ is the transport of 1,100 bricks per day, as derived by the Mahatma Gandhi Labour Institute, Ahmedabad. We included the wages received per 1,100 bricks to assess whether employers are adhering to this stipulation.

#### Qualitative data analysis

Deductive coding was conducted to identify responses relating to the topics of the research piece and to add greater depth to responses from the livelihood questionnaire. Inductive coding was also conducted to allow themes and sub-themes to emerge from the data. Coding was performed in several iterations, and new themes and sub-themes were noted wherever they appeared. The analysis was repeated until the coding was considered complete and no new themes emerged.

All semi-structured interview transcripts were analysed using thematic analysis in N-Vivo (V12.2, QSR International).

## Results

**Figure 1.**
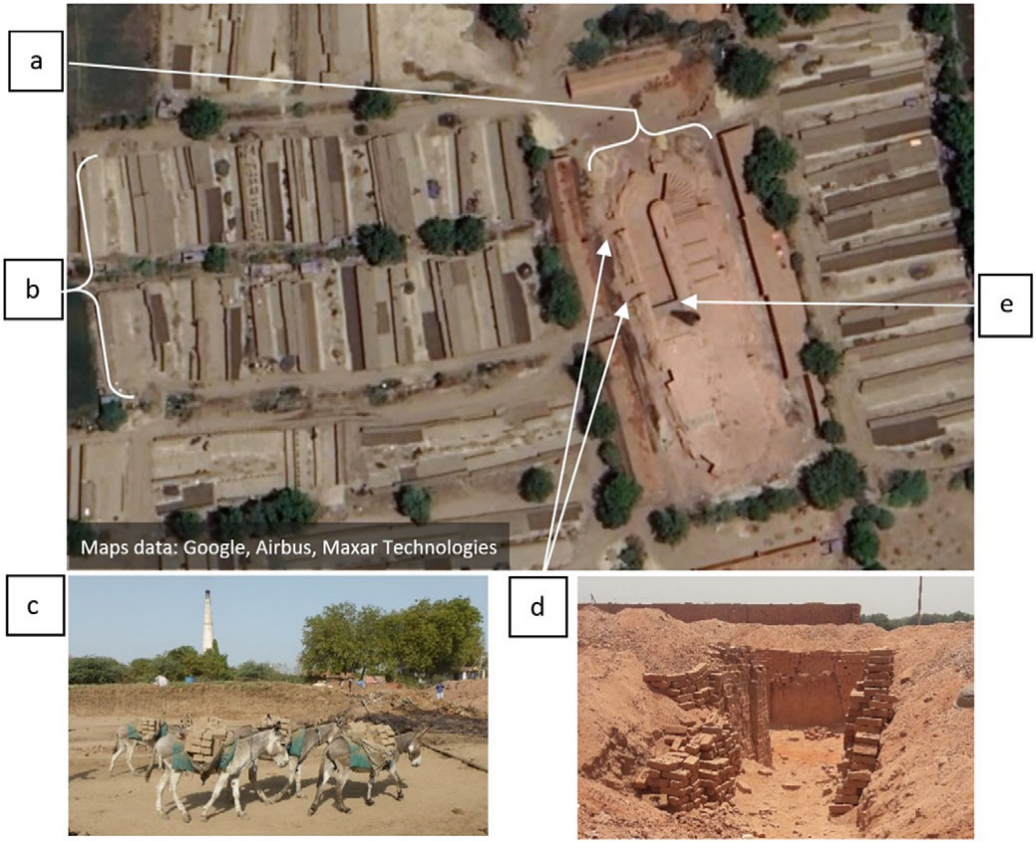
An example brick-kiln site visited during fieldwork in Gujarat, India, during May 2018, to demonstrate the movement of donkeys during brick transportation into the kiln (a). Showing wet bricks moulded and stacked in rows (b), where they are loaded into pack saddled carried by donkeys (c). Donkeys and their owners then walk to a kiln entrance (d), and through to where the bricks are offloaded and stacked within the kiln (e), ready to be covered and fired once the kiln is full. (Photo credit (c, d): LM Kubasiewicz).

All of the equids found in the kilns were donkeys, although brick kilns within Gujarat that employ mules were mentioned. None of the participants had knowledge of horses working in other kilns. Welfare assessments were completed for 220 donkeys in total, with a mean of six donkeys assessed per owner. Living conditions assessments were completed in all 14 kilns. Questionnaires were conducted with 37 donkey owners, five of which were thekedars, six non-owners and three kiln owners. Of these, 28 donkey owners, including four thekedars, and six non-owners completed semi-structured interviews. The movement of donkeys and owners during brick transportation is presented in Figure 1.

### EARS assessment results

According to the donkey owners (including thekedars), the main welfare problems they encountered were lameness (89% of responses; n = 33), with six donkey owners specifically mentioning lameness due to thorns; wounds from harnesses and bites from other donkeys (32%; n = 12), and colic (30%; n = 11).

As donkeys were usually tethered during welfare assessments, it was not always possible to assess donkeys for lameness. However, 49% (n = 112) of donkeys had overgrown hooves and 28% (n = 63) had hooves which were chipped or cracked. Ninety-eight percent (n = 214) of donkeys in the cohort had scars, alopecia, swellings or scabs, with forty-two percent of these (n = 93) having open wounds at the time of assessment. Where it was possible to identify a cause, wounds were predominantly due to bites from other donkeys and poorly fitting harnesses, confirming owners’ assertions, as well as from beating by donkey owners in order to encourage donkeys to walk or to increase their speed. Beating by owners was observed by researchers in almost half of the kilns in the cohort (n = 6), where the behaviour tended to be carried out by the majority of donkey owners, or none, within a particular kiln. Owners were not observed interacting with their donkeys in all kilns, however, so it is possible that this practice occurred elsewhere.

Over half of the donkeys (54%; n = 118) displayed signs of fear and distress, including sudden ‘startle’ responses when standing quietly (n = 48), head shyness (n = 33) and unpredictable or sudden movements (n = 18). Sixty percent of donkeys also had a negative response to the assessor when approached, including 40% (n = 87) that either turned their head, or whole body, away from the assessor.

#### Linking human and donkey behaviour

Rough handling was observed in 54% of cases where the donkey responded negatively to observer approach, as opposed to 17% where observer approach elicited a positive response. As recorded in field notes:

*Kiln A: “Owners were very rough in handling their donkeys; one owner punched and kicked a resting donkey in the stomach to get it to stand. Donkeys were difficult to assess, all very nervous, backing away, head shy and one tried to kick me when assessing.”*

*Kiln B: “Saw donkey owners interacting with donkeys; gentle, resting on the donkeys and when letting them off hobbles used soft clicking and ‘ush ush.’ No other signs of physical contact, e.g., slap or push. Very few donkey bites here, donkeys seemed much more relaxed.” [8^th^ May 2018]*

### Living conditions

Workers in all of the kilns resided in brick shelters with corrugated metal roofs. Two kilns contained permanent structures, including shelters for workers, food troughs for donkeys and areas for storing equipment (specifically harnesses) during the off-season, with all other kilns containing temporary structures. Water was collected from a single communal point or well provided by the kiln owner in all but two kilns. This water point was used for bathing and drinking water for both humans and donkeys. A single kiln had individual water pipes to each shelter home; whilst in one other kiln, workers were required to travel approximately 1 km to a point provided by a local farmer, as the water source provided by the kiln owner was not sufficient.

Shelter was available for donkeys in 29% (n = 4) of the kilns; elsewhere, donkeys were often tethered in the sun during the hottest part of the day (after finishing work). Where shelter was available, this was via trees close to the owners’ homes within the kilns; none of the donkeys had access to purpose-built shelters. Grazing areas were between 200 m and 2 km away from the kilns. Water was provided by donkey owners at set times and was not freely available to donkeys in any of the kilns.

### How do wages compare to established thresholds?

Mean daily wages for donkey owners calculated using piece rates, number of bricks moved per day (in total and per donkey) and number of donkeys worked per day, fell within 1 standard deviation of the totals stated directly by participants (mean [± SD] = Rs411 [± 105]; n = 4).

Piece rates were agreed verbally between each donkey owner and the thekedar prior to the brick-kiln season. Thekedars earned a mean of Rs187 (± 15); n = 3, and donkey owners earned a mean of Rs166 (± 14); n = 10 per 1,100 transported bricks. All kiln workers reported that they worked for seven days per week. A single thekedar had a family of two, with each adult earning Rs357 per day. For donkey owners, mean wages were Rs327 (± 122); n = 12 per adult per day.

**Figure 2.**
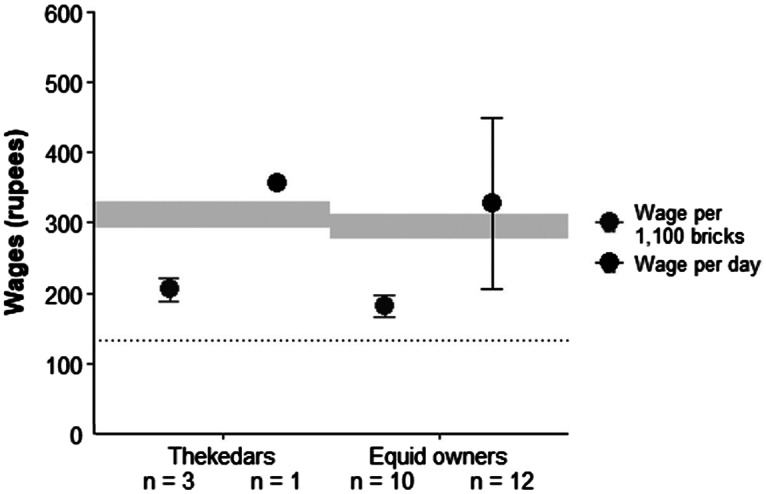
Average wages (per day per person), for thekedars and donkey owners working in the brick kilns in Ahmedabad, India. Error bars represent 1 standard deviation. The grey bar indicates the minimum wage range for the Brick Manufacturing Industry in Gujarat, for bhatiwala (Rs276.2 to 312.2) and supervisor (Rs293 to 329.2). The dotted line indicates the threshold wages for ‘extreme poverty’ set by the World Bank (Ferreira et al., 2015).

The thekedars’ wages were Rs28 higher than the state minimum, including special allowance, for skilled workers (Figure 2). Mean wages for donkey owners were Rs15 above the state minimum including special allowance, although wages varied widely, with the range including wages both above and below the state minimum (Figure 2).

Compared to the international poverty line (Rs133 per day), mean wages for thekedars and donkey owners were Rs224 and Rs194 above the minimum level, respectively (Figure 2).

### Bonded labour overview

All non-owners (100%; n = 6) and the majority of owners (97%; n = 37) received an advance payment from the kiln owner, via the thekedar, before the start of the kiln season. Thirty-seven percent of these owners used part of the advance to purchase at least some of the donkeys for the season. The majority used the rest of the advance money to pay for basic household expenses during the off-season, with fewer people using the money for medicines or for one-off events such as family weddings.

A kiln owner explained the process of advance payment during the semi-structured interviews. Equid owners in this particular kiln receive an advance during Diwali, the Hindu ‘festival of lights’, in October. Negotiations regarding the amount of advance take place individually with each kiln worker; these used to take place during the ceremonies of ‘Raksha Bandhan’ (a festival held during August) but have drifted back to Diwali over the years. To decide whether to employ people, the kiln owner reportedly looks at the donkeys, decides if the people have ‘good character’ and if they are able repay the advance (though did not specify how this is determined).

The kiln owner reported to providing advanced payment of, on average, Rs25,000 to Rs30,000 (or 20% of total earnings for one kiln season) per family, usually via the male head of household, although the amount can vary widely between families. Conversely, donkey owners that were willing to provide details of their advanced payments reported to have taken, on average, Rs50,000 to Rs70,000. Agreements were made verbally with no discernible contracts.

**Figure 3.**
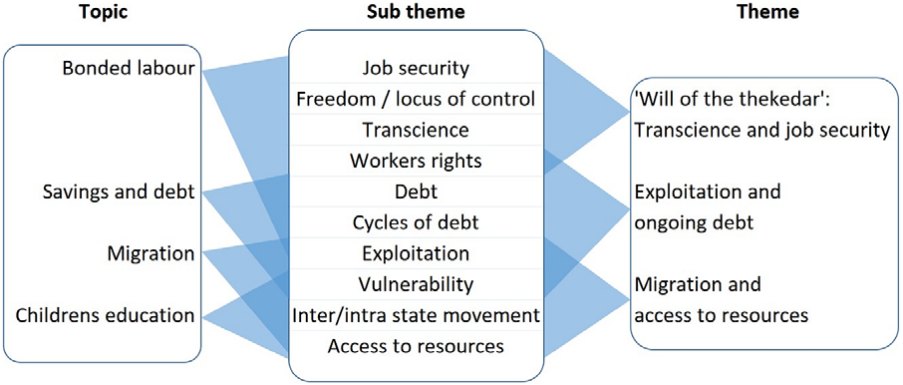
Overview of the sub-themes and themes that emerged from semi-structured interviews with participants in the brick kilns of Gujarat, India. Interviews covered the broad topics outlined below, but the direction of the interview was guided by the interests and views of the participant.

An overview of the themes identified from the data is given in Figure 3 and each one described below.

### The ‘will of the thekedar’: Transience and job security

The vast majority of respondents did not feel they had the freedom to seek work at different brick kilns, with respondents who felt they had the option to move limited to those with other stable sources of income (i.e. tractor driving). Although one donkey owner responded that they would “move to get the best price”, the kiln owner was present during this part of the interview, which may have influenced the response.

Much of the employment process is reliant upon the relationship between kiln owner or thekedar and worker, and the former’s opinion of the latter, with several workers reporting the need for a ‘good rapport’ and ‘trust’ between themselves and their employer. Many respondents reported that they would only receive an advance from an owner that they already knew or had worked with before, severely limiting their employment options. One donkey owner travelled over 1,000 km to work in a specific kiln and had been doing so for the previous four years:

*“The owner will give advance only to people who he knows, so that means we’ll have to come here only because the new owner will not give us the advance.”*

The lack of employment regulation, enforcement of equality laws or workers’ rights undoubtedly leads to discrimination and a lack of employment security, as recorded in field notes:

*“Participant [a donkey owner] is disabled, he is totally reliant on the will of the thekedar; during recruitment, donkey owners are offered specific routes along which to transport bricks for the entire season. If a route is too far for him, or if other kiln workers are available, this participant almost certainly won’t get the work. He mentioned that he had moved every few years because of this.” [9^th^ May 2018]*

Kiln owners did not demonstrate a duty of care towards workers, leaving workers with a constant sense of uncertainty and vulnerability, as demonstrated by field notes:

*“The atmosphere at the kiln was quite tense, people looked very tired and the donkeys were in particularly poor condition. It was coming towards the end of the season, with people preparing to leave. One worker told us that the kiln manager needed extra bricks to meet a quota, so was dismantling the brick shelters that the workers lived in to salvage the extra bricks. Despite this, the kiln owner had withheld the final payments to the workers for the last two days, forcing them to sleep out in the open, exposed and vulnerable.” [1^st^ May 2018]*

### Exploitation and ongoing debt

Exploitation was widely observed, with wages deducted disproportionately should workers not be able to meet their quotas:

*“This year [we came] fifteen, twenty days late because someone from our family got married. We had a loss because… the [kiln] owner took five lakh [500,000] bricks in a tractor. I can’t even ask advance for that, so loss is loss.”*

When asked if they would lose money, the respondent said that the 500,000 bricks would be deducted from their quota for the season. This amount of bricks was more than they could have transported in the twenty lost days, and far more than they could make up for in their remaining time at the kilns. The earnings from these bricks are subsequently lost to the family, who will need to repay a higher proportion of the advance in subsequent weeks, whilst having lost a disproportionate amount of their wages for the season. It was, however, unclear whether a formal arrangement was secured for the family to arrive late for the kiln season.

Two kiln owners reported to have moved away from using tractors or horse-and-cart systems towards using donkeys with panniers in order to reduce the number of brick breakages, with one stating that the breakages did not occur when donkeys were employed in this way. However, a proportion of the donkey owner’s wages, equating to 10%, was still deducted to account for expected breakages:

*“The owner says ‘you should do a hundred [bricks] more every day for each donkey.’ We do a thousand and then we do a hundred extra and they aren’t counted. He [the kiln owner] is saying ‘oh you may break some, some damage can happen. I pay you only a thousand but you’ll have to give one thousand one hundred.’ That means the moulders, the counters and everybody.”*

Conversely, some kiln owners have moved in the opposite direction, towards using tractors for moving bricks, or employing mule owners from Northern India for cart work in order to increase productivity. As a result, several of the donkey owners we interviewed had been displaced from the kiln they had worked in for the past three years. We were, however, unable to explore this in detail as the current kiln manager arrived at that point during the interviews and the respondents were reluctant to discuss the matter further.

According to a thekedar, people ‘occasionally’ work in different brick kilns and repay the advance via the thekedar at the new kiln, who will transfer the money to the previous kiln owner during the next Diwali or the following season (i.e., taking two seasons to pay back a single advance). This arrangement, however, depended on personal relationships between workers, thekedars and kiln owners, lacking any formal regulation. More often, both kiln owners and workers reported that advanced payments were reimbursed in full before the end of the current season.

This arrangement has the potential to create an inescapable spiral of debt for kiln workers, who live on the poverty line, making unexpected expenses impossible to budget for:

*“We lost two donkeys during the off season and had to borrow more money than we could repay during the kiln season to replace them.”*

Another respondent was waiting for her husband to return from visiting a money lender in the local town. The family had borrowed money from other donkey owners to purchase donkeys at the start of the season and had repaid this loan with the money earned during the kiln season. However, they did not have enough money remaining to repay the brick-kiln owner for their advance, and so had no choice but to take out a further loan to repay the advance, simply moving their debt from lender-to-lender. The visit was supposed to have taken less than a day, but the husband had been absent for three days, leaving his wife and young baby in a vulnerable situation, with the prospect of remaining alone at the kilns.

### Migration and access to resources

Of the thekedars, donkey owners and non-owners that discussed their travel to the kilns during semi-structured interviews, all lived within the kilns for the entire season. All thekedars (n = 3) and all donkey owners except one (n = 19) lived within the state of Gujarat during the off-season, with all thekedars and just over half of the donkey owners living within 100 km of the kilns. During SSIs, five equid owners in two kilns mentioned grouping together with other owners at the kiln to hire a truck to transport themselves and their equids to the kiln. At least three of these owners lived within 100 km of the kiln. All non-owners and a single donkey owner had travelled over 1,000 km from Uttar Pradesh. Although sample size is small for both thekedars and non-owners, the consensus among participants and local co-authors was that this trend is the norm, with non-donkey-owning kiln workers often travelling from more northern and western states such as Uttar Pradesh and Chhattisgarh.

There was a consensus among all non-owners that work was better paid in Gujarat than their home state and that more jobs were available. All of the moulders within the cohort also cited the warmer weather as a reason for travelling south, finding it very uncomfortable to mould the wet bricks in the colder conditions in Uttar Pradesh, whilst the firemen wanted to ‘avoid the tough winters.’

Donkey owners from further afield were not prevalent within the current cohort, and the majority of participants acknowledged that donkey movement is often prohibited across states due to the possible transmission of glanders.

#### Access to children’s education

All participants within the cohort, except for one donkey owner and two thekedars, had at least one school-age child. Children were observed working in the majority of the kilns visited, although fieldwork was conducted in the school holidays, so it is not possible to say with certainty whether all of these children worked during term time. Several participants said their children did work in the kilns, with some working after school and a small number acknowledging full-time work.

**Figure 4.**
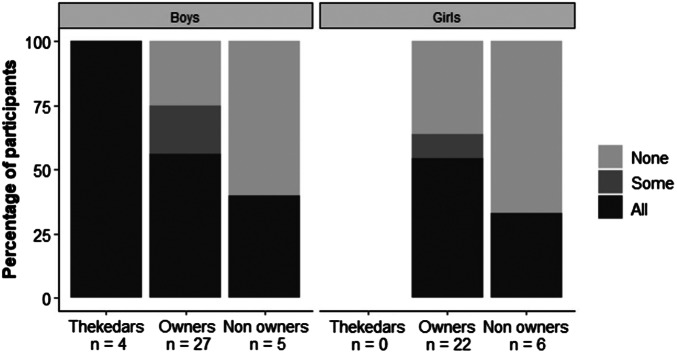
Percentage of parents who send all, some or none of their male and female children to school. Results are presented for each job role of the parents.

Fifty-five percent of the donkey owners sent all of their children to school (Figure 4); of these, 85 and 79%, respectively, sent their male and female children full time. Three thekedars reported to have male children only, all of whom attended school full time. Less than half of the non-owners sent their children to school (40% male, 33% female; Figure 4), all of whom attended full time.

Most of the donkey owners that discussed their children’s school attendance in detail had sought admission to a village school near the brick kilns during the season. A donkey owner that lived approximately 100 km away, in Balasinor, explained:

*“During the brick-kiln season the boy, he attends the school here, after the brick-kiln season they take their admission in the village school. It’s all connected, so they can easily transfer admission.…. Before they leave from here, the teacher of the school speaks to that particular teacher in the village. It’s a mutual understanding. Mutually they give their consent, and the child gets admission in the village.”*

Despite the presence of ‘kiln schools’ in some kilns, these were poorly utilised by the participants in the current cohort. One donkey owner reported to have sent their children to a kiln school, whilst another referred to these schools as ‘troublesome’ and preferred to leave their children at home with their grandparents during the kiln season to continue to attend their usual school.

Reasons for poor or no school attendance were mixed among donkey owners, with some citing a lack of interest from the child. Although responses were often similar for male and female children, three participants kept their female children at home ‘to help with household chores’, whilst none of the male children were kept home for this reason. A minority (n = 2) felt that their work in the brick kilns was the main factor preventing their children from attending school, due to a lack of attendance during the kiln season and subsequent inability to catch up once they had returned home. Both of these participants stated that they moved around too much for their children, who travelled with them, to attend school during the kiln season.

By contrast, all of the non-donkey-owners in the cohort reported difficulty in sending their children to school during the brick-kiln season. Two of these participants left their children at home with family so that they could stay at their local school, whilst the remaining four non-owners brought their children to the kilns with them, and none attended school. Migration was the predominant reason for this, as the owners were from a different state and said that they would need ‘ID proof’ to register their children. Language barriers were also acknowledged, along with the fact that the family migrate to the brick kiln part way through the school year and said they were not able to register their children at that time.

Whilst the vast majority of participants saw value in their children attending school, often citing hopes of them ‘getting a good government job’, in some cases the gain in productivity from child labour was thought to outweigh any benefits of schooling due to a lack of employment opportunities. One donkey owner, who had no education herself, stated:

*“What is the point of school, I send two sons to school to study but they don’t get any work in the government so what’s the point? Even if I made them go to school and study […] what’s the point when there is [sic] no jobs so better they work for us.”*

## Discussion

### Welfare and living conditions

Owners of working equids are under-represented in both literature and policy, with the majority of references to working conditions or wages omitting to mention the use of working animals. Donkey and human welfare are, however, intrinsically linked, with the poorly maintained hooves of donkeys mirroring the “raw and abraded hands” of their owners (Watson *et al.* 2020). Reactions of fear and distress were elicited more frequently from equids with aggressive owners, as previously seen for working mules (Ali *et al.* 2019; Norris *et al.* 2020) and demonstrated here for donkeys. Previous assessments of the current cohort found that owners who are less financially secure, specifically those on lower wages or who rely on loans, tend to own donkeys with poorer welfare in terms of behaviour (Kubasiewicz *et al.* 2022). Here, we add depth to that finding by suggesting that it is the owner’s behaviour itself which elicits such a response in their donkeys, potentially due to stress induced by their financial situation. Whilst the pressure to generate income may result in overworked, apathetic donkeys (Burn *et al.* 2010b), we propose that this pressure, coupled with workers own ill-treatment in the kilns, may manifest in mistreatment of their donkeys and produce behavioural responses associated with fear, distress and anxiety, as well as conspecific aggression.

Similarly, the living conditions of donkeys reflected that of their owners. Both humans and donkeys resided in small temporary shelters, with sparse protection from the elements and, in most cases, limited access to water. Donkey owners had very little autonomy within the kiln environment and kiln owners were often unsupportive if not actively exploitative. Intervention work from animal welfare and humanitarian aid organisations is, therefore, vital if the situation of equids and their owners is to improve. This support, however, must tackle the problem from all sides, acknowledging the intrinsic links between human and equid welfare, as well advocating for policy reform and enforcement.

### Wage and production

On average, donkey owners’ wages put them above the international poverty line during the brick-kiln season. In the vast majority of cases, however, these earning are required to cover at least part of the off-season, with all but one donkey owner receiving an advance for their work in the brick kilns for basic living expenses during the off-season and to purchase equids upon which they rely financially. Off-season work has been described as ‘sporadic and poorly paid’ by the current cohort (Kubasiewicz *et al.* 2022), increasing their reliance on, and potential exploitation by, the brick-kiln industry.

Donkey owners received the minimum daily wage set by the state government of Gujarat, but their output was far in excess of the ‘production norm’ of transporting 1,100 bricks during a working day. As there is no mention of working equids in the official government guidance for minimum wages, it is possible that production norms were set for workers without animals; this would, however, result in the loss of any advantage of equid ownership, particularly considering the cost of equid care, as it suggests that piece rates have been scaled down to account for the higher productivity of equid owners. The use of piece rate wages gives “tremendous scope for employers to manipulate the compensation package” (John 2014) as appropriate time-scales within which to complete the work are not provided or, as seems to be the case here, can be ignored. Lack of regulation in the brick kilns is well documented (Gupta 2003) and the use of both daily and piece rate figures gives licence to kiln owners to exploit the system, gaining far more from each worker than would be considered legal with adherence to daily production norms.

### Will of the thekedar: Transience and job security

The experience of debt-bondage by donkey owners appears to be similar to that of the other kiln workers discussed by Ercelawn and Nauman (2004), John (2014) and Anti-Slavery International (2017). Despite donkey owners describing the ‘trust’ required between kiln owners and workers, who return to the same kilns year-after-year, their treatment might not obviously reflect that of trusted and valued workers. Advanced payments have previously been described as a way to maximise exploitation of kiln workers through low wages, excessive hours and poor conditions (Breman 1996), with ‘trust’ previously described more accurately as ‘mutual suspicion’ (Guérin *et al.* 2007).

Bonded labour is heavily ingrained within the brick-kiln environment. Guérin *et al.* (2007) argue that as brick-kiln workers themselves are in favour of the bonded labour system due to necessity, poverty, and fear, simply advocating for its abolishment in the short term would not benefit those most vulnerable to its exploitation. Rather, a series of actions, emphasising social empowerment and contractual agreements, as well as potentially regulating advanced payments through external organisations, would at least lessen the current levels of corruption until permanent solutions arise. Donkey owners often exhibit perceived lack of control over their own fate (Brizgys 2018; Kubasiewicz *et al.* 2022); tackling this notion through social empowerment is likely to foster long-term resilience by providing people with the knowledge and confidence to negotiate their rights as employees.

### Exploitation and ongoing debt

Donkey owners are responsible for all costs associated with their donkeys, as well as losses associated with sickness or death of a donkey. Despite this, neither the increase in production associated with donkey ownership, nor the financial risks involved, are reflected in their wages nor in any employment law associated with the brick kilns. Although kiln workers are classified as ‘skilled workers’ in some reports (John 2014; Anti-Slavery International 2017), references to skill level are generally sparse, with no mention of the skill level required for donkey handling. The problems associated with this lack of acknowledgement, coupled with the perpetual cycle of debt in which donkey owners find themselves as a result of the debt-bondage arrangement, are unlikely to be solved without considerable reform of the minimum wage act, an increase in regulation and enforcement of those regulations. Anti-Slavery International (2017) advocates for the use of a time-based payment system for brick moulders. Whilst we agree with this assertion, time-based rates must take into account the added financial burden and risk associated with donkey ownership, as well as the increased production made possible by their work.

Despite similar piece-rate wages for all donkey owners in the cohort, levels of production and, therefore, daily wages, varied widely. Previous research highlights child labour within the kilns (Anti-Slavery International 2017), as well as long working hours. Whilst most kiln workers in the current cohort work similar hours, we observed children working in most of the brick kilns in the study. There was, however, a lack of certainty by the donkey owners about their exact wages; wages were decided through informal verbal agreements, rather than formally documented, and donkey owners experienced very little clarity on the terms of their employment. *Ad hoc* deductions for perceived ‘breakages’, for example, were not contracted nor fully explained. Whilst the equivalent loss of wages, equating to a 10% deduction, or ‘katoti’, falls within previously reported limits of 5% in South India (Guérin 2014) and 15% in Pakistan (Ercelawn & Nauman 2004), in none of these reports, nor the current study, were actual breakages quantified. This lack of transparency further increases the risk of exploitation to workers, who have to rely on their employer to keep track of their remuneration fairly and often feel cheated by the system (John 2014).

Brick kilns are considered part of the agricultural, rather than industrial sector, making them exempt from several regulations aimed at industry, such as the provision of pension funds (Guérin 2014), all but ensuring the long-term poverty of their workers. Illness, accidents, or cultural demands such as large wedding ceremonies, can deepen the level of debt held by a bonded individual or family, who rarely manage to save money during the kiln season (Bhukuth 2005). Bales (2004) describes the similar situation in Pakistan as a ‘trap of poverty’ (ibid; p 194) which, in this case, was exacerbated by sexual abuse, low profitability of the kiln business, and religious and ethnic discrimination, as well as subsistence wages. This debt was highlighted by donkey owners who, through the loss or illness of their donkeys, can end up in dire circumstances. Women and children often inherit debt from male family members in India should they decide to leave, with cases of both being sold into marriage and prostitution by land-owners when a bonded male employee leaves the land in Pakistan (Human Rights Watch 1995).

### Migration and access to education

Despite the fact that brick-kiln employees tend to be seasonal, migrant workers, the debt-bondage arrangement ensures a steady return to the kilns year-after-year. This arrangement leaves people somewhere between casual and permanent employees, allowing kiln owners to ignore the provisions for both (Gupta 2003). As opposed to non-donkey-owning kiln workers, however, donkey owners within the current cohort only migrated intra-state for work. Whilst donkey owners still experienced the pitfalls of bonded labour and poor working conditions, these intra-state migrants may have retained access to health and education services more easily than their inter-state colleagues (Wolfston 2019).

Although most donkey owners lived within Gujarat state during the off-season, almost half of those that discussed their home location travelled over 100 km to the kilns, with one donkey owner travelling from Uttar Pradesh. Whilst few participants discussed transport options, those that did mention it grouped together to hire trucks for themselves and their equids. Transport conditions are often poor for equids, with reports of wounds, fractures and dehydration in transit, as well as severe fear and distress (Weeks *et al.* 2012; Mitra & Valette 2017; Padalino & Riley 2020).

In previous studies, kiln workers have identified schools and crèches as the material provision they most need (Gupta 2003). In 2009–10, the Government initiative ‘Sarva Shiksha Abhiyan’ (Education for All) worked in conjunction with Prayas, a local humanitarian NGO, to pilot an education programme in the brick kilns. These schools were poorly attended, however, with only a basic syllabus and lack of progression for older children (Reed 2012). Language barriers were noted as an impediment to attendance for migrant children. Despite the majority of participants expressing a desire for their children to receive an education, school drop-out rates for migrant children remained high as children find it difficult to re-integrate into the education system outside of the working season (Reed 2012). Whilst barriers certainly still exist for intra-state migrant and transient workers to ensure children’s education, their children have the option to register at a local school, increasing their chances of receiving a full education.

### Animal welfare implications

Equids working in the Indian brick kilns support communities living in a perpetual state of debt. As such, donkey welfare is unlikely to improve without outside intervention (Ali *et al.* 2015). Support programmes must, however, focus on the underlying causes of poor welfare, taking into account the unique cultural traits of each community (Watson *et al.* 2020), as well as the links between human and donkey behaviour. Donkeys respond subtly to pain or discomfort so indicators of a problem can easily be missed (Regan *et al.* 2014), placing them in a particularly vulnerable position in the brick-kiln environment, where socio-economic pressures may take precedence.

## Conclusion

Humans and equids alike have been described as ‘invisible workers’ in the brick kilns (Valette 2015; Anti-Slavery International 2017), with both being neglected in policy and practice. The mental and physical conditions of donkeys mirror that of their owners, and this shared suffering between humans and their donkeys highlights the need for a one welfare approach to intervention work aimed at improving the health and welfare of donkeys and humans. These interventions, however, must cover policy reform to acknowledge the unique position of casual, migrant workers and the role of equids in supporting them. The considerable financial risks involved with donkey ownership, as well as the increased production that donkeys enable must be reflected in the policies aimed at protecting their owners.

Several strategies have been suggested to improve the human situation in the brick kilns including the collaboration of public bodies, NGOs and trade unions; formalisation of the employment and bonded labour process to aid regulation; and the social empowerment of workers (Guérin *et al.* 2007; Anti-Slavery International 2017). We propose that support for healthy working donkeys, and recognition of their role in policy and legislation relating to remuneration, working conditions and employee rights within the brick kilns are pivotal factors to the success of these strategies and ultimately for achieving acceptable standards of welfare for both donkeys and owners.

## Supplementary material

For supplementary material accompanying this paper visit https://doi.org/10.1017/awf.2023.1. click here to view supplementary material
