# Supplementary material for: Bonded labour and donkey ownership in the brick kilns of India: A need for reform of policy and practice
Source: Anim Welf. 2023 Jan 27;32:e8. doi: 10.1017/awf.2023.1 (PMC10936255; doi:10.1017/awf.2023.1)
Supplement: Supplementary file 1 [file S0962728623000015sup001.pdf]

Supplementary material

Table S1 EARS assessment carried out on equids working in brick kilns in Gujarat, India. The assessment formed part of a more comprehensive assessment for a wider research piece; only those relevant to the current research piece have been presented.

Question		Response options
Name of the location		*Open text*
Assessor name		*Open text*
Participant ID		*Numeric*
Does the participant consent to participating in this survey? (ask owner)		Yes; no
What are the main problems affecting the animals, according to the owners? (ask owner)		*Open text*
How did you acquire the donkeys you are using for this season? (ask owner)		Used my advance from the Thekedar; Borrowed money (alternative money lender); Used my savings; Other (please specify)
Is the equid lame?		*Yes (Severely lame / unable to walk / laid down/unable to raise); Yes (Lame but still moving); No lameness; Unknown*
Does the equid have any signs of hoof neglect or disease?		*None; Overgrown; Conformation defects; Chipped / cracked; Other (please specify); Unknown*
Are there any signs of integumentary system (skin or hair) alterations?		*No; Yes, open wounds; Yes, scars; Yes, alopecia; Yes, sarcoids; Yes, swellings; Other (please specify); Unknown*
In your opinion, what is the apparent cause of the lesions?		*Open text*
Please indicate signs of fear and distress present		*No signs of fear and distress present; Showing the whites of the eyes; Unpredictable or sudden movements; Sudden 'startle' responses when standing quietly (rapidly lifting the head); Aggressive behaviour; Trembling; Head shyness; Completely withdrawn/shut down; Other (please specify); Unknown*
Response to observer approach?	Positive / neutral	*Friendly approach (turning head towards the observer); Does not move*
	Negative	*Moves head away from observer; Moves whole body away when observer approaches; Aggressive behaviour towards observer - flattens ears, attempts to bite or kick*
	Unknown	*Unknown*
Owner's/user's/handler's interaction when holding the equid?	Positive / neutral	*Stroking; Light Touch; Resting against*
	Negative	*Rough interaction - slapping, hitting, yanking*
	Unknown	*No interaction; Unknown*

Table S2 Living conditions assessment carried out in brick kilns in Gujarat, India, to ascertain whether both human and equids basic needs are being met. Assessments were carried out with the assistance of participants when required.

Question	Response options
**People**	
Please describe the type of shelter available for people	*Open text*
Are these shelters temporary or permanent?	*Temporary; Permanent*
Please describe the water points available for people	*Open text*
How far is the nearest water point from where people reside?	*Open text*
Is water freely available?	*Yes; no*
**Equids**	
Please describe type of shelter available for equids	*Open text*
Are these shelters temporary or permanent?	*Temporary; Permanent*
Please describe the water points available for equids	*Open text*
How far is the nearest water point from the equids shelter / resting area?	*Open text*
Is water freely available?	*Yes; no*
Are the equids hobbled or loose at rest?	*Loose; Hobbled*
Distance to grazing	*Numeric (meters)*

Table S3   Questionnaire carried out with participants working in brick kilns in Gujarat, India. The questionnaire formed part of a more comprehensive questionnaire for a wider research piece; only those relevant to the current research piece have been presented.

Question	Response options
Name of the location	*Open text*
Assessor name	*Open text*
Participant ID	*Numeric*
Do you consent to participating in this survey?	Yes; no
**Demographic information**	
How old are you?	Under 18 (stop survey); Eighteen to Thirty; Thirty to Fifty; Over 50; Over 18, prefer not to give specific age
Gender	Male; Female
What is your job role in the kiln?	Brick loader (using a donkey); Brick loader (using a vehicle); Brick loader (using a hand cart); Brick moulder; Fireman; Brick stacker; Brick counter; Arranger; Unloader; Thekedar; Kiln manager; Kiln owner; Other (please specify)
How many adults (18 and over) are in your immediate household?	*Numeric*
**Wages and production**	
How much does your household earn in the brick kiln per week (in rupees) in total?	*Numeric*
How may days per week do you work?	*Numeric*
How much do you earn per 1000 bricks (made / transported)?	*Numeric*
How many bricks do you make / transport per day?	*Numeric*
How many bricks does each donkey transport per day?	*Numeric*
**Bonded labour (advance payments)**	
Were you paid an advance for your work in the kiln before the start of the season?	Yes; No; Unsure
How much were you paid for this advance?	*Numeric*
**Childrens' education**	
How many male children (under 18) are there in your household?	*Numeric*
How many female children (under 18) are there in your household?	*Numeric*
Do the school-age boys in your household go to school?	*Numeric*
How often do the boys attend?	*Numeric*
Do the school-age girls in your household go to school?	*Numeric*
How often do the boys attend?	*Numeric*

Table S4 A sample of the semi-structured interview questions carried out with participant working in the brick kilns in Gujarat, India. As interviews were semi-structured, specific questions differed between interviews, not all of the listed questions were asked during all interviews and follow-up questions were based on the responses and interests of the participant.

Topic	Participant	Question
Background	All	Can you tell me a bit about yourself and your family?
	All	How long have you worked in brick kilns?
	All	Do all of the family members that came here with you work in the kiln too? What do they do?
	Donkey owners	How long have you worked with donkeys?
	All	Why did you starting working in the brick kilns originally?
Bonded labour	All	What was the process for you to be recruited to work in the brick kiln this season?
	Kiln owners / thekedars	Could you explain the employment process / how you find employees?
	All	Could you explain how / when you are paid for your work?
Savings and debt	Kiln workers	Do you save any money from your work in the brick kilns?
	Kiln workers	Do you have any other sources of income / work?
Migration / Living conditions	Kiln owners / thekedars	Could you tell us about your home during the kiln season / off season?
	Kiln workers	How far do you travel to the kiln? Why is that?
Childrens' education	All (with school age children)	How important is it that your child(ren) attend school?
	All (with school age children)	Where do(es) you child(ren) attend school?
	All (with school age children)	What are you hopes for your childrens future?
